# Association between body mass index and cardiopulmonary function in patients with chronic coronary syndrome

**DOI:** 10.1080/07853890.2026.2739133

**Published:** 2026-09-28

**Authors:** He Huang, Gaonian Zhao, Su Jiang, Jiangwei Ma

**Affiliations:** a Department of Rehabilitation, The Affiliated Taizhou People’s Hospital of Nanjing Medical University, Taizhou School of Clinical Medicine, Nanjing Medical University, Taizhou, Jiangsu Province, China; b Department of Cardiology, Fengxian District Central Hospital, Shanghai, China

**Keywords:** Chronic coronary syndrome, obesity paradox, cardiopulmonary exercise testing, body mass index, sex differences

## Abstract

**Background: **Patients with chronic coronary syndrome (CCS) are frequently complicated by reduced exercise tolerance. Previous studies exploring the relationship between body mass index (BMI) and cardiopulmonary function among CCS patients have reported conflicting results, and the definite association between the two indicators remains unclear. Accordingly, we performed this study to characterize their actual correlation.

**Methods: **This retrospective study examined the association between BMI and Cardiopulmonary Exercise Testing (CPET) indices and the modifying effect of sex in 405 CCS patients recruited from the Cardiovascular Disease Center of Taizhou People’s Hospital. Patients were divided into three groups based on BMI tertiles, and CPET parameters were compared. Multiple linear regression and sex-stratified analyses were conducted.

**Results: **In patients with CCS, BMI exerted a bidirectional linear differential effect on cardiopulmonary exercise capacity without nonlinear inflection points: higher BMI correlated with greater PeakVO_2_ (*B* = 22.851, 95%CI: 16.180–29.522, β = 0.270, *p* < 0.001), yet lower Peak VO_2_/kg (B=-0.214, 95%CI: −0.310–-0.117, β=-0.212, *p* < 0.001). This association was modified by sex. The positive association between BMI and PeakVO_2_ was stronger in males (*B* = 31.708, 95%CI: 21.609–41.806, β = 0.377, *p* < 0.001), whereas the negative inhibitory effect of BMI on Peak VO_2_/kg was more pronounced in females (B=-0.242, 95%CI: −0.370–-0.115, β=-0.271, *p* < 0.001). Moreover, other determinants of cardiopulmonary function exhibited sex disparities.

**Conclusion: **In patients with CCS, higher BMI was positively correlated with absolute peak exercise capacity but negatively correlated with peak VO_2_/kg, and this correlation showed sex heterogeneity.

## Introduction

Coronary Artery Disease (CAD) is the leading cause of increased mortality and frequent adverse cardiovascular events worldwide, and its disease spectrum has gradually shifted from acute coronary syndrome to Chronic Coronary Syndrome (CCS) [1]. In the CCS phase, although patients may have preserved ventricular systolic function, the comorbidity of metabolic disorders and cardiac dysfunction is extremely prevalent [2]. This not only significantly impairs patients’ quality of life but also imposes a heavy socioeconomic burden on the healthcare system, manifested as repeated hospitalizations, increased demand for revascularization procedures, and rising costs of long-term pharmacological management [3].

Currently, a comprehensive system for the clinical diagnosis and treatment of CAD has been established, including lifestyle intervention, intensive pharmacotherapy, Percutaneous Coronary Intervention (PCI), and Coronary Artery Bypass Grafting (CABG) [4]. However, even under standardized treatment, some patients still face persistent declines in exercise tolerance and poor long-term prognosis [3]. Existing risk prediction factors for exercise, such as BMI and lipid profiles, have demonstrated limited predictive accuracy for an individual’s exercise tolerance and functional outcomes because they fail to consider the various complex cardiopulmonary physiological states of these people. Therefore, cardiopulmonary exercise tests can be used to assess the level of exercise tolerance for individuals more precisely, and offer scientific support for changes in clinical practice [5,6].

According to published epidemiological data and previous studies, the relationship between obesity and heart disease is not obvious and may even be adverse [7]. Other kinds of diseases that are easy to get from being overweight or obese include high blood pressure, diabetes and high blood cholesterol (hyperlipidemia), etc. On the other hand, the ‘obesity paradox’ phenomenon has been frequently reported in cohorts of heart failure, chronic kidney disease and some coronary heart disease patients; that is to say, people with a high BMI show a lower mortality rate and better survival prognosis [8].

Some studies have shown that in some obese individuals, the risk of serious heart and blood vessel disease does not rise with an increase in BMI, and some patients may even be doing better under this condition.At present, there are different explanations for the mechanism of the obesity paradox. Some scholars believe that the contradiction may be due to an increase in the energy-storage capacity, muscle mass or inflammatory factors [9]. Recently, some studies have found that among obese patients with heart failure and reduced ejection fraction, peak oxygen consumption increases with an increase in BMI; however, it is not directly related to reduced mortality [10].

Most of the current research focuses on how obesity affects the end points of cardiovascular diseases (such as survival rate and rehospitalisation rate), yet few cross-sectional studies have delineated the cross-sectional correlations between BMI and detailed cardiopulmonary function parameters. We aimed to clarify whether BMI presents divergent correlations with cardiopulmonary exercise indicators among patients with chronic coronary syndrome. Accordingly, this retrospective cross-sectional study analysed the correlational patterns and potential influencing factors between BMI and cardiopulmonary exercise testing measurements in patients with coronary artery disease.

## Materials and methods

### Research design and subjects

#### Chronic coronary syndrome

The diagnosis of CCS in this study was formulated in accordance with the 2019 European Society of Cardiology (ESC) Guidelines for the Diagnosis and Management of Chronic Coronary Syndromes. CCS is defined as persistent imbalance between myocardial oxygen supply and demand caused by organic coronary artery stenosis, coronary vasospasm or coronary microvascular dysfunction. Its clinical manifestations include stable angina pectoris, silent myocardial ischemia, post-acute coronary syndrome (ACS) recovery phase, and stable status after coronary revascularization. Patients were required to be free of ACS episodes or acute heart failure (AHF) exacerbation within 1 month prior to admission and maintain long-term clinical stability.

#### Cardiopulmonary exercise testing

Cardiopulmonary exercise testing (CPET) was performed on an electromagnetically braked cycle ergometer using a symptom-limited incremental exercise protocol. Anaerobic threshold (AT) was identified by the V-slope method. Peak respiratory exchange ratio (peak RER) was documented to evaluate test maximality. A sensitivity analysis confined to participants with peak RER ≥ 1.05 was implemented to assess whether submaximal exercise altered the study findings (Supplementary File 1). Breath-by-breath data needed to compute VE/VCO_2_ slope and OUES were not available in the present dataset.

#### Fat-free mass

Fat-free mass (FFM) is defined as total body mass excluding adipose tissue, which primarily consists of skeletal muscle, bone, visceral organs and body fluid. Fat-free mass was estimated from anthropometric measurements using validated James equations:

$$\text{For men}:\text{ FFM}=\text{1.10}\times \text{Weight}-128\times ({\text{Weight}}^{2}/{\text{Height}}^{2})$$

$$\text{For women}:\text{ FFM}=\text{1.07}\times W-148\times ({\text{Weight}}^{2}\text{​}/{\text{Height}}^{2})$$

We adopted estimated FFM to normalize peak VO_2_ and re-examine the association between adiposity and exercise capacity. This approach eliminates inherent mathematical coupling between BMI and peak VO_2_/kg.

This study was conducted in accordance with the ethical principles of the Declaration of Helsinki, and approved by the Ethics Committee of Taizhou People’s Hospital (LSKY 2026-020-01). We retrospectively collected clinical data of CCS patients visiting the Cardiovascular Center from January 2024 to December 2025. After enrollment and screening, 405 patients were included in the study (Figure 1).

**Figure 1.**
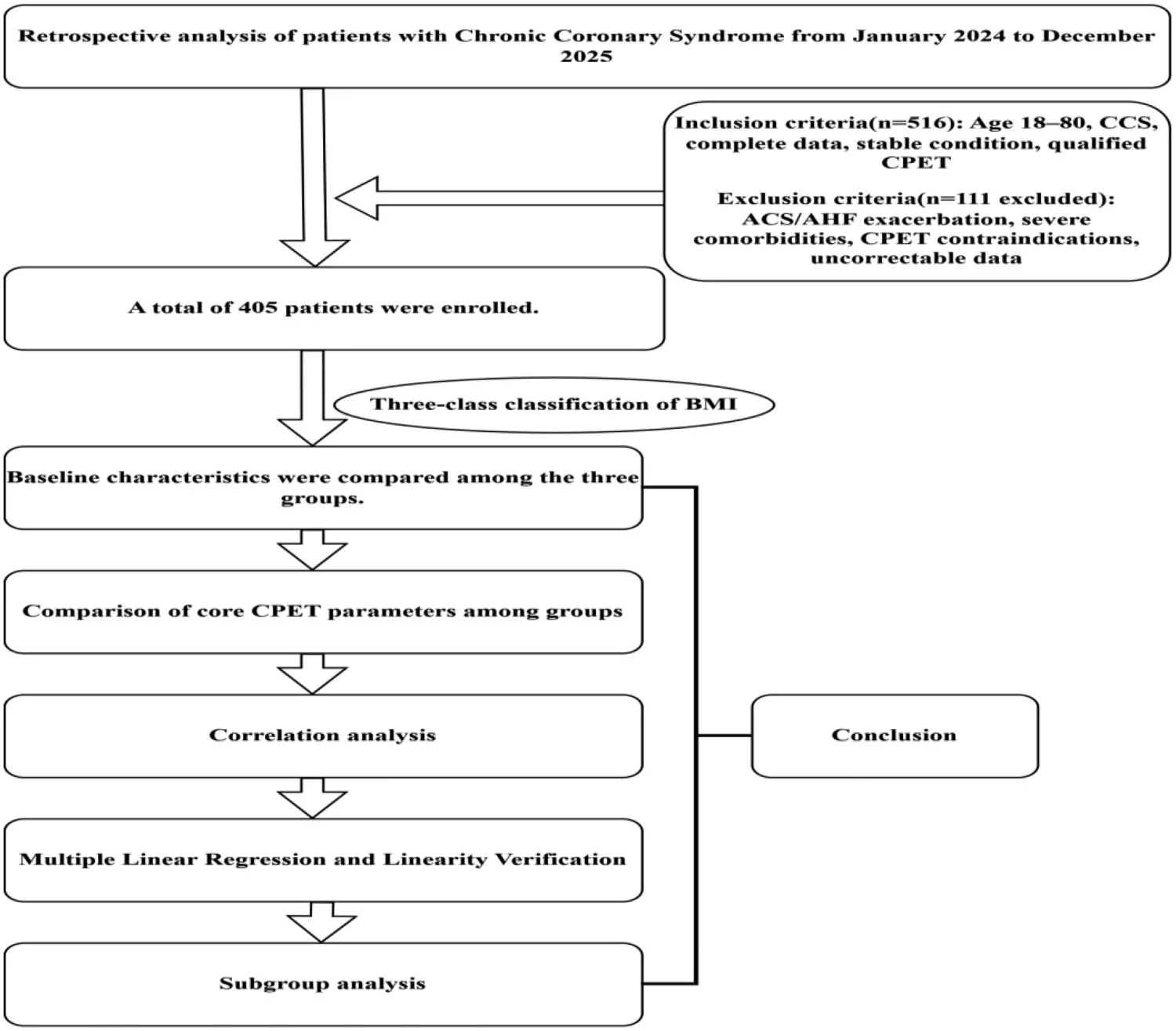
Flow chart. BMI, body mass index; CCS, Chronic Coronary Syndrome; ACS, Acute Coronary Syndrome; CPET, Cardiopulmonary Exercise Testing.

Inclusion Criteria:

- Aged between 18 and 80 years and clinically diagnosed with CCS;
- Complete clinical records, including basic information such as age, sex, height, weight, and BMI;
- Completed standardized CPET with successful acquisition of complete parameters including anaerobic threshold (AT), oxygen uptake (VO_2_), and exercise load;
- Clinically stable with no acute cardiovascular events in the past 3 months;
- Complete and traceable clinical data and laboratory test results.

Exclusion Criteria:

- Diagnosed with acute coronary syndrome (including ST-segment elevation myocardial infarction, non-ST-segment elevation myocardial infarction, and unstable angina) or experiencing an acute heart failure exacerbation (n = 19);
- Complicated with severe systemic diseases such as severe pulmonary disease, severe valvular heart disease, severe arrhythmia, end-stage renal disease, or malignant tumor (n = 27);
- Having contraindications to exercise and unable to complete adequate cardiopulmonary exercise testing (n = 13);
- Exercise limitation due to skeletal muscle diseases, severe osteoarthropathy, or neurological disorders (n = 21);
- Missing key clinical data, incomplete test indicators, or obviously abnormal data that cannot be corrected (n = 7);
- Recent severe infection, surgery, trauma, or cachectic state (n = 24).

#### Statistical power

The total sample size of this study was 405 patients, including 237 males and 168 females, and a priori statistical power evaluation was conducted in accordance with the statistical requirement for multivariate regression models (events per predictor variable, EPV ≥ 10); the overall multivariate regression model included 14 clinical independent variables, and the total sample of 405 patients supported stable model fitting with statistical power reaching the standard of ≥0.8, the interaction model incorporating the sex*BMI interaction term retained a total of 14 independent variables with unchanged overall sample size and consistent statistical power compared with the main-effect model to effectively identify the independent interactive effect, while sex-stratified multivariate regression analyses adopted 13 independent variables for the male subgroup (*n* = 237) and female subgroup (*n* = 168) respectively, in which the male subgroup had sufficient sample size and favorable model statistical power, whereas the relatively small sample size of the female subgroup slightly reduced the power to detect weak effect sizes.

#### Statistical analysis

The 405 enrolled patients with CCS were divided into low, medium, and high BMI groups according to BMI tertiles rather than conventional international BMI cut-off values. This tertile grouping ensured balanced sample sizes in three subgroups to stabilize intergroup comparisons and regression analysis. Differences in demographic characteristics, clinical baseline data, laboratory examination indicators, and CPET parameters were compared among the three groups. A correlation matrix heatmap was used to visually demonstrate the correlations between BMI and various CPET parameters. FFM was introduced for comparative correlation analysis to verify that the associations between BMI and CPET parameters were not merely derived from the inherent structural formula of BMI. Confounders for the regression models were selected based on clinical relevance and univariate screening. No mathematical transformation was performed on any continuous variables prior to regression modelling. Multiple linear regression models were established to assess the independent impact of BMI on exercise tolerance after adjustment for all confounding covariates: height, sex, age, smoking history, hypertension (HTN), diabetes mellitus (DM), glycated hemoglobin (HbA1c), triglyceride (TG), high-density lipoprotein cholesterol (HDL-C), hemoglobin (Hb), left ventricular ejection fraction (LVEF), history of β-blocker use, and prior revascularization. Restricted cubic spline regression with four knots was applied to further validate the dose–response nonlinear trend. In addition, the interaction term of BMI × Sex was incorporated into the regression model to test interaction effects and preliminarily identify sex heterogeneity in the BMI-exercise tolerance association, followed by sex-stratified regression analyses.

Missing data were assessed *via* Little’s MCAR test. Variables with missingness >20% were removed, and multiple imputation was used for remaining missing data. All statistical analyses were performed using SPSS version 27.0 (IBM Corp., Armonk, NY, USA). The normality of continuous variables was assessed using the Shapiro-Wilk test. Normally distributed continuous variables were presented as mean ± standard deviation (SD), and intergroup comparisons were performed using one-way analysis of variance (ANOVA). Non-normally distributed continuous variables were expressed as median (interquartile range, IQR), and intergroup differences were compared using the Kruskal-Wallis H test. Categorical variables were described as number (percentage, %), and group comparisons were performed using the Chi-square test. Linear regression and restricted cubic spline dose–response nonlinear analyses were conducted in R.

The primary objective was to examine the independent association between BMI and cardiopulmonary exercise capacity, quantified by peak VO_2_ and peak VO_2_/kg as two co-primary endpoints. To account for multiplicity, a Bonferroni correction was applied to these primary multivariable regression analyses, with statistical significance set at *p* < 0.025 (0.05/2). Nonlinearity was assessed as a precondition for the primary linear models; these analyses were considered supplementary and not separately powered. Correlation analyses with other CPET parameters, interaction analyses (sex × BMI), and sex-stratified analyses were predefined as exploratory and were performed without formal multiplicity adjustment. Sex-stratified analyses were conducted only to explore a potential interaction effect; given the exploratory nature, these results should be interpreted with caution.

## Results

### Baseline characteristics

A total of 405 subjects were enrolled in this study and stratified into three groups based on BMI tertiles. Intergroup comparison prominently highlighted baseline imbalances primarily in age (*p* = 0.048), sex (*p* < 0.001), weight (*p* < 0.001), height (*p* = 0.002), smoking (*p* = 0.002), HTN (*p* < 0.001), DM (*p* = 0.028) and FFM (*p* < 0.001). Additional significant intergroup differences were also observed in HbA1c (*p* = 0.021), TG (*p* < 0.001), HDL-C (*p* < 0.001) and Hb (*p* < 0.001), with all these variables showing *p* < 0.05. In contrast, thyroid function indicators (FT3, FT4, TSH), TC, LDL-C, LVEF, β-blocker use, and Revascularization showed no statistically significant intergroup differences (all *p* > 0.05) (Table 1).

**Table 1.** Baseline characteristics of patients with chronic coronary syndrome according to BMI tertiles.

Characteristics	Total *n* = 405	BMI Q1(17.63-23.44)21.67 ± 1.32 *n* = 137	BMI Q2(23.44-26.04)24.86 ± 0.77 *n* = 134	BMI Q3(26.04-39.34)29.00 ± 2.68 *n* = 134	*p*
Age, years	59(52,66)	61(54, 68)	58(52, 65)	57(49, 66)	0.048
Height, cm	166(160,171)	163(158, 170)	167(160, 172)	168(162, 172)	0.002
Weight, kg	69.5(60.0, 76.0)	58.0(54.0, 63.0)	69.7(63.5,74.0)	80.0(75.0, 85.5)	<0.001
Sex, male %	58.5	46.0	62.7	67.2	<0.001
Smoking, %	24.2	13.9	27.6	31.3	0.002
HTN, %	63.5	48.9	66.4	75.4	<0.001
DM, %	20.5	14.6	19.4	27.6	0.028
FT3, pmol/L	4.87(4.41, 5.33)	4.77(4.28, 5.32)	4.87(4.37, 5.30)	5.03(4.49, 5.45)	0.146
FT4, pmol/L	10.61(9.699, 12.08)	10.60(9.77, 12.07)	10.87(9.91, 12.12)	10.40(9.49, 11.63)	0.142
TSH, pmol/L	1.85(1.18, 2.66)	1.77(1.17, 2.70)	1.97(1.20, 2.68)	1.78(1.18, 2.59)	0.796
HbA1c, %	6.00(5.70, 6.50)	5.90(5.65, 6.40)	6.10(5.75, 6.40)	6.20(5.75, 6.70)	0.021
TC, mmol/L	4.05(3.33, 4.95)	4.15(3.45, 5.07)	4.07(3.18, 4.85)	4.02(3.37, 4.66)	0.316
TG, mmol/L	1.19(0.84, 1.76)	0.98(0.70, 1.07)	1.21(0.89, 1.77)	1.50(1.07, 2.07)	<0.001
HDL-C, mmol/L	1.12(0.95, 1.33)	1.29(1.06, 1.49)	1.10(0.95, 1.24)	1.03(0.90,1.20)	<0.001
LDL-C, mmol/L	2.53(2.01, 3.17)	2.52(2.00, 3.26)	2.51(1.89, 3.17)	2.54(2.09, 3.10)	0.828
LVEF, %	70(65, 73)	70(65,73)	70(65,73)	70(65,73)	0.425
Hb, g/L	134(125, 146)	129(122,139)	137(129,146)	140(128,152)	<0.001
β-blocker use, %	28.9	29.9	32.8	23.9	0.256
Revascularization, %	31.9	29.2	35.1	31.3	0.576
FFM, kg	51.67(44.23, 57.91)	43.84(40.45, 51.82)	53.34(44.66, 57.59)	57.91(49.67, 61.25)	<0.001

BMI, body mass index; HTN, hypertension; DM, diabetes mellitus; FT3, free triiodothyronine; FT4, free thyroxine; TSH, thyroid-stimulating hormone; HbA1c, glycated hemoglobin A1c; TC, total cholesterol; TG, triglycerides; HDL-C, high-density lipoprotein cholesterol; LDL-C, low-density lipoprotein cholesterol; LVEF, left ventricular ejection fraction; Hb, hemoglobin; FFM,Fat-free mass.

### CPET characteristics

Significant differences in CPET were observed across different BMI groups. The higher BMI group demonstrated significantly higher absolute Peak VO_2_, AT,Max O_2_ Pulse, V_T, FVC and FEV_1_ compared with the lower BMI group. In contrast, body weight-adjusted relative oxygen uptake efficiency indices, including Peak VO_2_/kg, RAT, and Max MET, were significantly lower in the higher BMI group. These findings suggest that elevated BMI is associated with higher absolute exercise capacity, but reduced cardiopulmonary efficiency per kilogram of body weight (Table 2).

**Table 2.** Differences in cardiopulmonary parameters across BMI tertiles.

Characteristics	Total *n* = 405	BMI Q1(17.63-23.44)21.67 ± 1.32 *n* = 137	BMI Q2(23.44-26.04)24.86 ± 0.77 *n* = 134	BMI Q3(26.04-39.34)29.00 ± 2.68 *n* = 134	*p*	η²(95%CI)
Peak VO₂, mL/min	923(774, 1165)	842(692,1007)	931(792,1214)	1100(857,1312)	<0.001	0.1050(0.053, 0.162)
Peak VO₂/kg, mL/(kg·min)	14.1(12.0, 16.5)	14.3(12.3,16.7)	13.9(12.0,17.2)	13.4(11.3,15.9)	0.017	0.023(0.001, 0.057)
Peak VCO₂, mL/min	899(718, 1196)	775(642,948)	935(726,1287)	1075(797,1325)	<0.001	0.076(0.032, 0.127)
AT, mL/min	866(716, 1085)	763(637,902)	870(730,1080)	968(819,1203)	<0.001	0.109(0.057, 0.167)
RAT, mL/(kg·min)	12.75(10.91, 14.66)	13.27(11.45,15.12)	12.51(10.76,14.86)	12.69(10.51,14.05)	0.020	0.022(0.001, 0.056)
Max MET, METs	3.6(3.1, 4.2)	3.7(3.2,4.3)	3.6(3.1,4.2)	3.6(3.0,4.0)	0.041	0.019(0.001, 0.051)
Max O₂ Pulse, mL/beat	8.7(7.0, 10.3)	7.5(6.4, 9.0)	9.1(7.4, 10.4)	9.5(8.1, 11.9)	<0.001	0.052(0.016, 0.097)
RR, breaths/min	16.12(14.17, 18.47)	16.30(14.44, 18.98)	15.87(13.95, 17.47)	16.75(14.23, 18.90)	0.092	0.012(0.000,0.039)
MVV, L/min	49.21(35.10, 65.83)	47.57(34.77, 66.20)	48.78(34.64, 65.83)	51.29(37.39, 64.64)	0.685	0.005(0.000, 0.023)
V_T, L	0.55(0.43, 0.68)	0.46(0.39, 0.59)	0.57(0.46, 0.70)	0.60(0.49, 0.76)	<0.001	0.094(0.045,0.149)
FVC, L	2.96 ± 0.85	2.76 ± 0.80	3.00 ± 0.86	3.12 ± 0.85	0.002	0.031(0.005, 0.069)
FEV₁, L	2.42 ± 0.72	2.27 ± 0.70	2.45 ± 0.71	2.53 ± 0.74	0.010	0.023(0.001, 0.057)
ΔVO₂/ΔW, mL/min/W	10.74(9.45, 12.22)	10.67(9.68,12.18)	10.58(9.01,12.12)	11.01(9.50,12.85)	0.150	0.009(0.000,0.034)
Peak RER	1.06 ± 0.14	1.05 ± 0.14	1.08 ± 0.14	1.05 ± 0.12	0.113	0.013(0.000, 0.041)

BMI, body mass index; Peak VO_2_, peak oxygen uptake; Peak VO_2_/kg, peak oxygen uptake per kilogram; Peak VCO_2_, peak carbon dioxide output; ΔVO_2_/ΔW, Change in oxygen uptake per increment of work rate; AT, anaerobic threshold; RAT, relative anaerobic threshold; Max MET, maximal metabolic equivalent; Max O_2_ Pulse, maximal oxygen pulse; MVV, maximal voluntary ventilation; V_T, tidal volume; RR, respiratory rate; FVC, forced vital capacity; FEV_1_, forced expiratory volume in one second; Peak RER, peak respiratory exchange ratio.

### Correlations of BMI and FFM with cardiopulmonary function parameters

Correlation matrix heatmap analysis clearly showed that BMI was significantly associated with multiple cardiopulmonary functional indicators in patients with coronary artery disease. Specifically, BMI was significantly positively correlated with absolute exercise tolerance and lung volume indicators including Peak VO_2_, AT, Max O_2_ Pulse, V_T, FVC and FEV_1_. In contrast, BMI was significantly negatively correlated with weight-adjusted relative oxygen efficiency indicators such as Peak VO_2_/kg, RAT and MET. Among these pairwise correlations, associations between BMI and Peak VO_2_, Peak VO_2_/kg, Peak VCO_2_, AT, Max O_2_ Pulse, V_T, FVC, and FFM reached statistical significance at *p* < 0.001, whereas associations between BMI and RAT, MET, and FEV_1_ reached statistical significance at *p* < 0.01. No statistically significant correlations were observed between BMI and ΔVO_2_/ΔW, MVV, or RR. FFM was correlated with Peak VO_2_ (*r* = 0.16, *p* < 0.01), and showed a significant negative correlation with Peak VO_2_/kg (r=-0.18, *p* < 0.001) (Figure 2).

**Figure 2.**
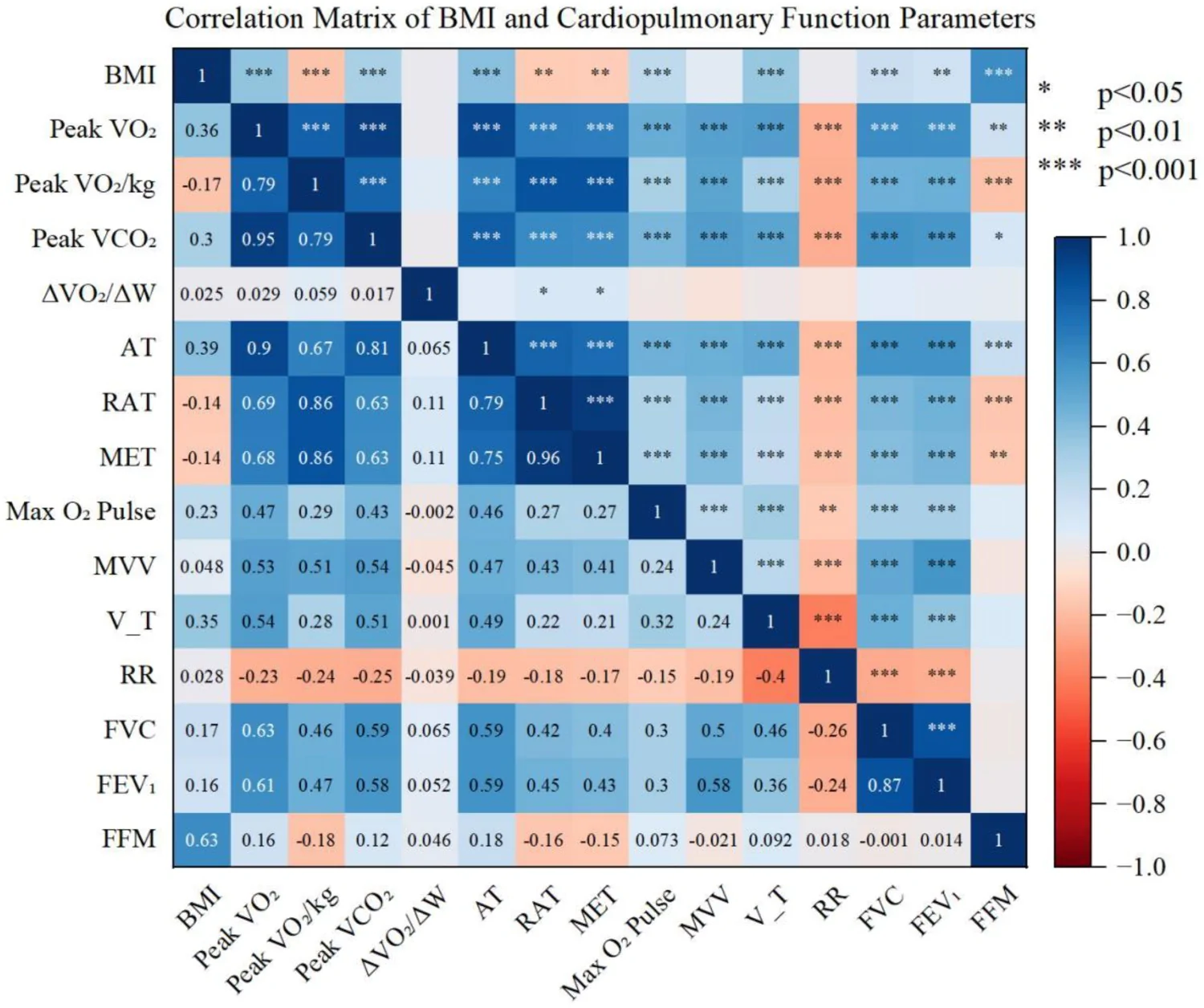
Correlation matrix heatmap. BMI, body mass index; Peak VO_2_, peak oxygen uptake; Peak VO_2_/kg, peak oxygen uptake per kilogram; Peak VCO_2_, peak carbon dioxide output; ΔVO_2_/ΔW, Change in oxygen uptake per increment of work rate; AT, anaerobic threshold; RAT, relative anaerobic threshold; Max MET, maximal metabolic equivalent; Max O_2_ Pulse, maximal oxygen pulse; MVV, maximal voluntary ventilation; V_T, tidal volume; RR, respiratory rate; FVC, forced vital capacity; FEV_1_, forced expiratory volume in one second; FFM, Fat-free mass Note: Parameters were divided into five categories: (1) Body composition indicators: BMI, FFM; (2) Peak aerobic metabolism indicators: Peak VO_2_, Peak VO_2_/kg, Peak VCO_2_, ΔVO_2_/ΔW; (3) Endurance indicators: AT, RAT, MET; (4) Cardiac function indicator: Max O_2_ Pulse; (5) Pulmonary ventilation function indicators: MVV, V_T, RR, FVC, FEV_1_.The following variable pairs have mathematical derivation relationships: Peak VO_2_ & Peak VO_2_/kg, Peak VO_2_ & Peak VCO_2_, AT&RAT&MET, FVC & FEV_1_.

### Multiple Linear Regression analysis

Multiple Linear Regression Analysis After adjusting for confounding factors including sex, age, height, smoking history, HTN, DM, TG and HDL-C, Hb, LVEF, β-blocker use, and revascularization status, multiple regression analysis showed that BMI was independently positively correlated with absolute Peak VO_2_ (*B* = 22.851, 95%CI: 16.180–29.522, β = 0.270, *p* < 0.001) in patients with CCS, but independently negatively correlated with Peak VO_2_/kg (B=−0.214, 95%CI: −0.310–0.117, β=−0.212, *p* < 0.001). The variance inflation factors (VIF) of all variables were less than 3, indicating no significant multicollinearity in the regression models. These findings further confirm the complex association between BMI and cardiopulmonary exercise function (Table 3).

**Table 3.** Multiple linear regression models for peak VO_2_ and peak VO_2_/kg.

	PeakVO₂(mL/min)					PeakVO₂/kg (mL/kg/min)				
Variables	B	95%CI	β	*p*	VIF	B	95%CI	β	*p*	VIF
BMI	22.851	16.180, 29.522	0.270	<0.001	1.346	−0.214	−0.310, −0.117	−0.212	<0.001	1.346
Height	8.869	4.959, 12.779	0.230	<0.001	2.241	−0.048	−0.104, 0.009	−0.104	0.097	2.241
Sex	−172.578	−241.255, −103.901	−0.287	<0.001	2.834	−2.624	−3.616, −1.631	−0.366	<0.001	2.834
Age	−7.421	−9.598, −5.244	−0.257	<0.001	1.235	−0.104	−0.135, −0.072	−0.301	<0.001	1.235
Smoking	−10.311	−63.781, 43.160	−0.015	0.705	1.293	−0.084	−0.856, 0.689	−0.010	0.832	1.293
HTN	−21.169	−66.652, 24.314	−0.034	0.361	1.189	−0.152	−0.809, 0.505	−0.021	0.65	1.189
DM	1.307	−31.895, 34.509	0.004	0.938	1.750	0.092	−0.388, 0.572	0.021	0.706	1.750
HbA1c	−20.200	−44.044, 3.644	−0.078	0.097	1.841	−0.287	−0.632, 0.058	−0.093	0.102	1.841
TG	−35.769	−62.653, −8.885	−0.100	0.009	1.223	−0.532	−0.921, −0.144	−0.125	0.007	1.223
HDL-C	114.897	29.770, 200.024	0.110	0.008	1.438	1.876	0.646, 3.107	0.150	0.003	1.438
Hb	2.388	0.722, 4.053	0.127	0.005	1.699	0.032	0.008, 0.056	0.143	0.009	1.699
LVEF	5.181	1.858, 8.504	0.111	0.002	1.091	0.072	0.024, 0.120	0.129	0.003	1.091
β-blocker use	−49.872	−98.209, −1.535	−0.076	0.043	1.192	−0.839	−1.538, −0.140	−0.108	0.019	1.192
Revascularization	−6.148	−56.540, 44.243	−0.010	0.811	1.368	−0.087	−0.815, 0.641	−0.012	0.814	1.368

BMI, body mass index; Peak VO_2_, peak oxygen uptake; Peak VO_2_/kg, peak oxygen uptake per kilogram; HTN, hypertension; DM, diabetes mellitus; HbA1c, glycated hemoglobin A1c; TG, triglycerides; HDL-C, high-density lipoprotein cholesterol; LVEF, left ventricular ejection fraction; Hb, hemoglobin.

Residuals had a mean of 0, with standardized residual ranging from −2.453 to 3.863 (Peak VO_2_) and −2.262 to 4.881 (Peak VO_2_/kg).

### Nonlinear relationship test

Restricted cubic spline regression with four knots was applied to evaluate the adjusted dose–response associations between BMI and two CPET parameters, including absolute Peak VO_2_ and Peak VO_2_/kg. After adjusting for confounding factors, a positive association between BMI and absolute Peak VO_2_ was observed, and the test for nonlinearity was not statistically significant (P for nonlinear = 0.915). In contrast, BMI exhibited a negative association with Peak VO_2_/kg, while no significant nonlinear relationship was detected likewise (P for nonlinear = 0.629) (Figure 3).

**Figure 3.**
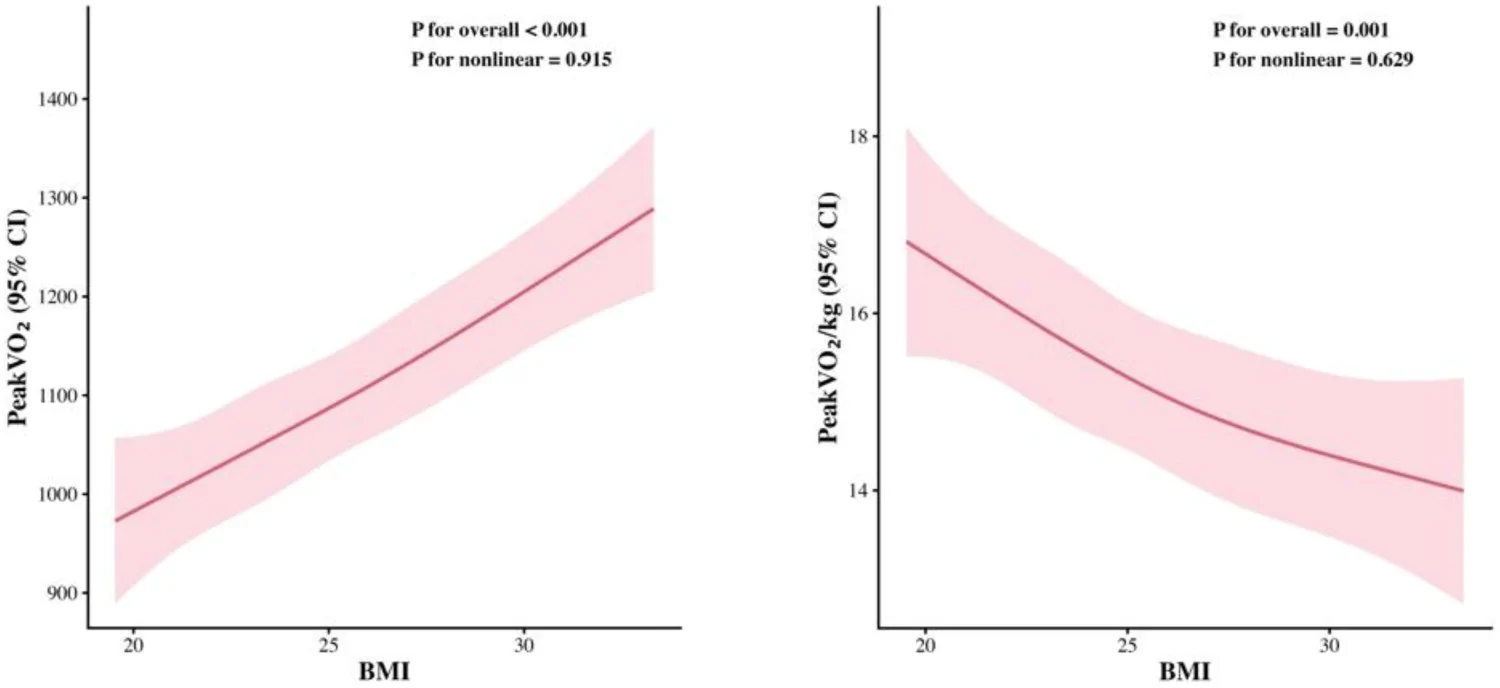
Adjusted dose–response associations between BMI, Peak VO_2_, and Peak VO_2_/kg. Note: Restricted cubic spline regression with 4 knots. Left panel: Absolute Peak VO_2_; Right panel: Peak VO_2_/kg. The solid red line shows the fitted relationship, and the shaded area represents the 95% confidence band.

## Sex stratification analysis

To explore whether sex modifies the effect of BMI on Peak VO_2_ and Peak VO_2_/kg in patients with CCS, multiple linear regression models were first constructed. Covariates including BMI, age, height, smoking status, hypertension, diabetes mellitus, glycated hemoglobin, triglycerides, high-density lipoprotein cholesterol, hemoglobin, left ventricular ejection fraction, β-blocker use, and revascularization were adjusted for, and the sex-by-BMI interaction term (Sex × BMI) was incorporated. The variance inflation factor (VIF) of all variables in the models was less than 3, indicating no obvious multicollinearity. In the model with Peak VO_2_ as the dependent variable, BMI was an independent positive influencing factor (*B* = 26.035, 95%CI: 19.232–32.837, β = 0.307, *p* < 0.001). When Peak VO_2_/kg served as the dependent variable, BMI exerted an independent negative effect (B=-0.170, 95%CI: −0.268–-0.071, β=-0.168, *p* < 0.001). Importantly, the Sex × BMI interaction term reached statistical significance in both regression models (*p* < 0.001), suggesting that the effects of BMI on Peak VO_2_ and Peak VO_2_/kg are modified by sex (Table 4).

**Table 4.** Predictors of peak VO_2_ and peak VO_2_/kg: multiple linear regression incorporating sex*BMI interaction.

	PeakVO₂(mL/min)					PeakVO₂/kg (mL/kg/min)				
Variables	B	95%CIa	β	*p*	VIF	B	95%CI	β	*p*	VIF
BMI	26.035	19.232,32.837	0.307	<0.001	1.406	−0.170	−0.268,−0.071	−0.168	<0.001	1.406
Height	8.902	5.062,12.741	0.231	<0.001	2.173	−0.041	−0.097,0.015	−0.089	0.151	2.173
Age	−7.287	−9.447,−5.127	−0.252	<0.001	1.222	−0.101	−0.132,−0.069	−0.292	<0.001	1.222
Smoking	−9.043	−62.399,44.314	−0.013	0.739	1.294	−0.092	−0.867,0.683	−0.011	0.815	1.294
HTN	−19.772	−65.134,25.590	−0.032	0.392	1.189	−0.130	−0.789,0.529	−0.018	0.697	1.189
DM	0.269	−32.878,33.417	0.001	0.987	1.753	0.088	−0.393,0.570	0.020	0.719	1.753
HbA1c	−19.884	−43.655,3.887	−0.077	0.101	1.839	−0.296	−0.641,0.049	−0.096	0.093	1.839
TG	−34.735	−61.557,−7.914	−0.097	0.011	1.224	−0.518	−0.908,−0.129	−0.121	0.009	1.224
HDL-C	116.914	31.963,201.865	0.112	0.007	1.439	1.867	0.633,3.101	0.150	0.003	1.439
Hb	2.219	0.542,3.895	0.118	0.010	1.730	0.031	0.007,0.056	0.139	0.012	1.730
LVEF	5.190	1.877,8.502	0.111	0.002	1.091	0.071	0.023,0.119	0.128	0.004	1.091
β-blocker use	−51.662	−99.88,−3.443	−0.079	0.036	1.192	−0.864	−1.565,−0.164	−0.111	0.016	1.192
Revascularization	−5.419	−55.633,44.795	−0.009	0.832	1.365	−0.062	−0.791,0.667	−0.008	0.867	1.365
Sex*BMI	−7.069	−9.769,−4.369	−0.293	<0.001	2.737	−0.100	−0.139,−0.06	−0.347	<0.001	2.737

BMI, body mass index; Peak VO_2_, peak oxygen uptake; Peak VO_2_/kg, peak oxygen uptake per kilogram; HTN, hypertension; DM, diabetes mellitus; HbA1c, glycated hemoglobin A1c; TG, triglycerides; HDL-C, high-density lipoprotein cholesterol; LVEF, left ventricular ejection fraction; Hb, hemoglobin.

Residuals had a mean of 0, with standardized residual ranging from −2.463 to 3.935 (Peak VO_2_) and −2.238 to 5.000 (Peak VO_2_/kg).

To further interpret the statistically significant Sex*BMI interaction effect, sex-stratified multiple linear regression analyses were performed in the present study. In the Peak VO_2_ model, BMI was independently and positively correlated with Peak VO_2_ in both males (*B* = 31.708, 95%CI: 21.609–41.806, β = 0.377, *p* < 0.001)) and females (*B* = 15.108, 95%CI: 6.996–23.219, β = 0.265, *p* < 0.001), with a larger effect magnitude observed in the male group. In the Peak VO_2_/kg model, BMI exerted a significant negative effect in females (B=-0.242, 95%CI: −0.370–-0.115, β=-0.271, *p* < 0.001), whereas the negative association of BMI was relatively weaker in males (B=-0.156, 95%CI: −0.299–-0.014, β=-0.149, *p* = 0.031).

Beyond BMI, there were evident sex-based disparities in other independent predictors of peak oxygen uptake. For Peak VO_2_: height, HbA1c, HDL-C, and LVEF were independent influencing factors only in males; TG, Hb, and β-blocker use showed statistical associations only in the female group. For Peak VO_2_/kg: height exerted an independent effect only in the female group; HDL-C was associated with Peak VO_2_/kg only in males;Hb, TG, and β-blocker use were significantly correlated with Peak VO_2_/kg only in the female group. Stratified regression analyses indicate that the determinants of Peak VO_2_ and Peak VO_2_/kg exhibit marked sex heterogeneity among patients with CCS (Table 5).

**Table 5.** Stratified multiple linear regression analysis of predictors for peak VO_2_ and peak VO_2_/kg by sex.

Variables	Male					Female				
Peak VO₂	B	95%CI	β	*p*	VIF	B	95%CI	β	*p*	VIF
BMI	31.708	21.609,41.806	0.377	<0.001	1.468	15.108	6.996, 23.219	0.265	<0.001	1.261
Height	11.375	5.887, 15.864	0.218	<0.001	1.123	4.737	−0.450, 9.923	0.122	0.073	1.112
Age	−8.246	−11.343, −5.148	−0.297	<0.001	1.262	−6.083	−8.964, −3.321	−0.309	<0.001	1.339
Smoking	−19.785	−81.711, 42.140	−0.033	0.530	1.092	99.582	−52.604, 251.768	0.087	0.198	1.096
HTN	6.023	−61.914, 73.960	0.010	0.861	1.232	−42.231	−98.791, 14.329	−0.106	0.142	1.263
DM	8.653	−33.799, 51.105	0.026	0.861	1.600	1.489	−52.031, 55.009	0.005	0.956	2.216
HbA1c	−39.485	−73.498, −5.472	−0.149	0.023	1.680	−7.052	−39.346, 25.242	−0.043	0.667	2.449
TG	−31.090	−66.948, 4.767	−0.093	0.089	1.166	−47.708	−86.317, −9.099	−0.185	0.016	1.390
HDL-C	212.389	81.526, 343.252	0.186	0.002	1.334	26.215	−74.311, 126.740	0.040	0.607	1.486
Hb	0.764	−1.404, 2.931	0.040	0.488	1.294	4.810	2.082, 7.538	0.240	<0.001	1.152
LVEF	7.243	3.048, 11.439	0.176	<0.001	1.061	−1.801	−7.409, 3.806	−0.043	0.527	1.118
β-blocker use	−54.642	−121.987, 12.704	−0.088	0.111	1.195	−97.248	−166.108, −28.388	−0.212	0.006	1.407
Revascularization	6.675	−62.847, 76.196	0.011	0.85	1.381	−1.594	−70.113, 66.925	−0.003	0.963	1.293
**Peak VO₂/kg**										
BMI	−0.156	−0.299, −0.014	−0.149	0.031	1.468	−0.242	−0.370, −0.115	−0.271	<0.001	1.261
Height	−0.021	−0.098, 0.056	−0.0322	0.595	1.123	−0.095	−0.176, −0.013	−0.155	0.024	1.112
Age	−0.114	−0.158, −0.071	−0.329	<0.001	1.262	−0.092	−0.138, −0.047	−0.299	<0.001	1.339
Smoking	−0.261	−1.134, 0.611	−0.035	0.556	1.092	1.474	−0.923, 3.871	0.082	0.226	1.096
HTN	0.178	−0.779, 1.136	0.023	0.714	1.232	−0.508	−1.399, 0.383	−0.081	0.262	1.263
DM	0.177	−0.442, 0.775	0.042	0.561	1.600	0.188	−0.655, 1.031	0.042	0.66	2.216
HbA1c	−0.569	−1.049, −0.090	−0.172	0.02	1.680	−0.098	−0.606, 0.411	−0.038	0.705	2.449
TG	−0.461	−0.966, 0.044	−0.110	0.074	1.166	−0.781	−1.389, −0.173	−0.192	0.012	1.390
HDL-C	2.978	1.133, 4.822	0.209	0.002	1.334	0.740	−0.844, 2.323	0.072	0.357	1.486
Hb	0.011	−0.019, 0.042	0.047	0.465	1.294	0.078	0.035, 0.121	0.248	<0.001	1.152
LVEF	0.100	0.041, 0.159	0.195	0.001	1.061	−0.015	−0.103, 0.073	−0.023	0.74	1.118
β-blocker use	−0.760	−1.709, 0.189	−0.098	0.116	1.195	−1.585	−2.670, −0.500	−0.220	0.004	1.407
Revascularization	0.041	−0.939, 1.021	0.005	0.935	1.381	−0.071	−1.150, 1.008	−0.009	0.897	1.293

BMI, body mass index; Peak VO_2_, peak oxygen uptake; Peak VO_2_/kg, peak oxygen uptake per kilogram; HTN, hypertension; DM, diabetes mellitus; HbA1c, glycated hemoglobin A1c;TG, triglycerides; HDL-C, high-density lipoprotein cholesterol; LVEF, left ventricular ejection fraction; Hb, hemoglobin.

Residuals had a mean of 0, with standardized residual ranging from −2.274 to 3.339 (male Peak VO_2_), −3.124 to 3.642 (female Peak VO_2_), −2.180 to 4.377 (male Peak VO_2_/kg) and −2.671 to 3.123 (female Peak VO_2_/kg).

## Discussion

This study enrolled 405 patients with chronic coronary syndrome after rigorous screening, utilizing cardiopulmonary exercise test data to objectively quantify their cardiopulmonary function. Through group comparisons, correlation analyses, multiple regression, and sex-stratified analyses. Results revealed that the high BMI group exhibited higher absolute peak oxygen uptake and other cardiopulmonary energy reserves, but lower relative peak oxygen uptake and relative anaerobic threshold. The association was found to be linear and influenced by sex.

BMI is the most widely used anthropometric indicator in epidemiological research owing to its simple measurement, low cost, and applicability to large-scale population screening. Compared with BMI, waist circumference (WC), waist-to-hip ratio (WHR), visceral adipose tissue (VAT), and body fat percentage (BF%) exhibit superior predictive performance for adverse clinical endpoints including major adverse cardiovascular events (MACE) [11]. WC and WHR directly reflect central obesity, a well-established risk factor for endothelial dysfunction and impaired oxygen transport capacity [12]. VAT quantified by imaging techniques has the strongest correlation with chronic cardiovascular stress and metabolic deterioration, while BF% can accurately evaluate the overall adiposity level in obese female patients with low muscle mass [13]. Even so, BMI remains irreplaceable for primary population health screening. Patients with elevated BMI have a higher prevalence of hypertension, diabetes mellitus, and hypertriglyceridemia yet demonstrate better exercise tolerance, indicating that BMI-related cardiovascular risk factors are not directly correlated with physical functional reserve [14]. Previous studies have verified that obese individuals maintain lean body mass through increased skeletal muscle mass and cardiac output to compensate for elevated metabolic demands, leading to higher absolute peak oxygen uptake (peak VO_2_) [15]; however, this compensatory adaptation disrupts glucose and lipid metabolism. In the present study, significant intergroup differences were only observed in triglyceride (TG) and high-density lipoprotein cholesterol (HDL-C), while total cholesterol (TC) and low-density lipoprotein cholesterol (LDL-C) showed no between-group differences. These findings suggest that obesity drives metabolic disease predominantly *via* abnormalities in triglyceride-rich lipoproteins rather than isolated cholesterol elevation, and such characteristic metabolic disorders further impair cardiopulmonary function [16]. The reduced peak VO_2_/kg in the high-BMI group may be attributed to the extra mechanical load required to mobilize excess adipose tissue, which increases oxygen consumption under an equivalent absolute workload [17]. Marked sex disparities were also observed at baseline: the high-BMI group contained a larger proportion of male participants with greater lean muscle mass than females, and muscle mass percentage was positively correlated with absolute physical function capacity [18,19]. We further explored the social and behavioural contributors to these sex-specific differences in physical function. Men are generally engaged in heavier manual labour and have higher rates of cigarette smoking and alcohol consumption [20]. Chronic cardiovascular stress induced by mechanical strain and other triggers may reduce maximal oxygen transport capacity in the setting of obesity. In contrast, female patients are more susceptible to joint disorders and burdens derived from social roles, making them more prone to a vicious cycle of physical deconditioning when complicated by obesity [21].

## Limitations

The present study has several inherent limitations. The single-center design may introduce regional selection bias, and fat-free mass was derived from predictive formulas instead of direct measurement, which may bring deviations from true values. Confounding variables such as statin medication, regular physical activity, and social determinants of health were not incorporated for adjustment. Besides, several participants could not reach maximal exertion during cardiopulmonary exercise testing, and the key parameters including the VE/VCO_2_ slope and OUES were unavailable for analysis. Moreover, an intrinsic mathematical association exists between BMI and weight-adjusted cardiopulmonary exercise variables, which may interfere with result interpretation. The statistical power of the dose-response analysis was limited in the high-BMI range, and the sample size for female subgroups in the stratified analysis was insufficient. Without long-term follow-up records, we cannot determine the predictive effect of absolute and relative cardiopulmonary functional reserve on patients’ long-term quality of life and survival. Future large-scale multicenter prospective cohort studies with imaging-based body composition measurement and prolonged follow-up will be conducted to establish high-quality evidence for clinical practice.

## Conclusion

This cross-sectional study systematically clarified the cross-sectional associations between BMI and cardiopulmonary exercise function in 405 patients with CCS: higher BMI was independently positively correlated with peak VO_2_, while independently negatively correlated with peak VO_2_/kg. These relationships exhibited linear features without nonlinear curves, and sex heterogeneity was observed in the cross-sectional correlations.

## Supplementary Material

Supplementary_File.docx

## Data Availability

All data can be requested from the corresponding author upon reasonable request.
