# Supplementary material for: Association between body mass index and cardiopulmonary function in patients with chronic coronary syndrome
Source: Ann Med. 2026 Sep 28;58(1):2739133. doi: 10.1080/07853890.2026.2739133 (PMC13629802; doi:10.1080/07853890.2026.2739133)
Supplement: Supplementary file 1 [file IANN_A_2739133_SM1618.docx]

### Supplementary File: 1

### For sensitivity analysis of principal components, we enrolled participants with peak respiratory exchange ratio (Peak RER)＞1.05. Statistical analyses included correlation analysis, multivariate regression analysis, and multivariate regression analysis incorporating the BMI*sex interaction term.

### Pearson Correlation Analysis Summary

#### 1. Correlations of Fat-Free Mass (FFM)

- **FFM2 vs. Peak VO₂ (mL/min)**: Significant positive correlation, r=0.244, **P<0.01**
- **FFM2 vs. Peak VO₂/kg**: Significant weak negative correlation, r=−0.140, **P=0.042 (P<0.05)**
- **FFM2 vs. BMI**: Strong positive correlation, r=0.659, **P<0.01**

#### 2. Correlations of Body Mass Index (BMI)

- **BMI vs. Peak VO₂ (mL/min)**: Moderate positive correlation, r=0.392, **P<0.01**
- **BMI vs. Peak VO₂/kg**: Significant moderate negative correlation, r=−0.197, **P=0.004 (P<0.01)**

Multivariate linear regression showed no obvious multicollinearity (all VIF＜3.0). BMI had inverse associations with two peak oxygen uptake indicators. BMI was positively correlated with absolute Peak VO₂ (B=28.799, 95%CI：18.794,38.803，β=0.330, P＜0.001) but negatively correlated with weight-normalized Peak VO₂/kg (B=−0.212, 95%CI：-0.358,-0.065，β=−0.207, P=0.005).

For Peak VO₂, height, sex, age, triglyceride (TG), left ventricular ejection fraction (LVEF) and coronary revascularization were significant influencing factors (P＜0.05). Smoking, hypertension (HTN), diabetes mellitus (DM), HbA1c, HDL-C, hemoglobin (Hb) and β-blocker use showed no statistical significance (P＞0.05).

For Peak VO₂/kg, sex, age, TG, LVEF and revascularization remained significant (P＜0.05), while height and other clinical variables had no independent effect (P＞0.05).

| Variables | Peak VO₂ (mL/min) | | | | | Peak VO₂/kg (mL/kg/min) | | | | |
| --- | --- | --- | --- | --- | --- | --- | --- | --- | --- | --- |
|  | B | 95%CI | β | P | VIF | B | 95%CI | β | P | VIF |
| BMI | 28.799 | 18.794,38.803 | 0.330 | ＜0.001 | 1.433 | -0.212 | -0.358,-0.065 | -0.207 | 0.005 | 1.433 |
| Height | 10.943 | 5.115,16.772 | 0.281 | ＜0.001 | 2.443 | -0.041 | -0.126,0.045 | -0.089 | 0.348 | 2.443 |
| Sex | -154.863 | -257.707,-52.02 | -0.243 | 0.003 | 2.843 | -2.519 | -4.027,-1.012 | -0.337 | 0.001 | 2.843 |
| Age | -5.598 | -8.793,-2.404 | -0.190 | ＜0.001 | 1.277 | -0.080 | -0.127,-0.033 | -0.231 | ＜0.001 | 1.277 |
| Smoking | -20.849 | -93.839,52.14 | -0.031 | 0.574 | 1.272 | -0.298 | -1.368,0.771 | -0.038 | 0.583 | 1.272 |
| HTN | 44.998 | -20.332,110.328 | 0.073 | 0.176 | 1.227 | 0.759 | -0.198,1.717 | 0.105 | 0.12 | 1.227 |
| DM | 26.276 | -26.186,78.739 | 0.064 | 0.324 | 1.783 | 0.366 | -0.403,1.135 | 0.076 | 0.349 | 1.783 |
| HbA1c | 7.798 | -33.29,48.885 | 0.026 | 0.709 | 2.060 | 0.127 | -0.475,0.729 | 0.036 | 0.678 | 2.06 |
| TG | -85.358 | -126.932,-43.784 | -0.240 | ＜0.001 | 1.496 | -1.193 | -1.803,-0.584 | -0.286 | ＜0.001 | 1.496 |
| HDL-C | -30.298 | -155.31,94.714 | -0.029 | 0.633 | 1.511 | -0.425 | -2.257,1.408 | -0.034 | 0.648 | 1.511 |
| Hb | 2.273 | -0.189,4.735 | 0.116 | 0.070 | 1.725 | 0.029 | -0.007,0.065 | 0.126 | 0.114 | 1.725 |
| LVEF | 6.422 | 1.285,11.559 | 0.125 | 0.015 | 1.092 | 0.086 | 0.011,0.161 | 0.142 | 0.026 | 1.092 |
| β-blocker use | -33.697 | -107.437,40.044 | -0.048 | 0.369 | 1.217 | -0.541 | -1.622,0.539 | -0.066 | 0.324 | 1.217 |
| Revascularization | -93.746 | -167.107,-20.385 | -0.150 | 0.013 | 1.510 | -1.389 | -2.464,-0.313 | -0.19 | 0.012 | 1.51 |

Multivariate linear regression analysis was performed, with all variance inflation factor (VIF) values less than 3.0 to exclude severe multicollinearity. BMI showed opposite independent correlations with two exercise capacity indicators: BMI was positively associated with absolute Peak VO₂ (B=30.487, 95%CI：20.391,40.583， β=0.349, P＜0.001), while it was negatively associated with weight-normalized Peak VO₂/kg (B=−0.186, 95%CI：-0.335,-0.038， β=−0.182, P=0.014). A significant **Sex×BMI interaction term** was detected for both outcomes (Peak VO₂: β=−0.234, P=0.004; Peak VO₂/kg: β=−0.306, P=0.003), indicating gender modified the association between BMI and peak oxygen uptake.

For Peak VO₂, height, age, triglyceride (TG), left ventricular ejection fraction (LVEF), coronary revascularization and Sex×BMI interaction were significant predictors (P＜0.05). Smoking, hypertension (HTN), diabetes mellitus (DM), HbA1c, HDL-C, hemoglobin (Hb) and β-blocker use had no significant effects (P＞0.05). For Peak VO₂/kg, age, TG, LVEF, revascularization history and Sex×BMI interaction remained statistically significant (P＜0.05), whereas height and other clinical covariates were not independently predictive (P＞0.05).

| Variables | Peak VO₂ (mL/min) | | | |  | Peak VO₂/kg (mL/kg/min) | | | |  |
| --- | --- | --- | --- | --- | --- | --- | --- | --- | --- | --- |
|  | B | 95%CI | β | P | VIF | B | 95%CI | β | P | VIF |
| BMI | 30.487 | 20.391,40.583 | 0.349 | ＜0.001 | 1.456 | -0.186 | -0.335,-0.038 | -0.182 | 0.014 | 1.456 |
| Height | 11.208 | 5.43,16.986 | 0.288 | ＜0.001 | 2.396 | -0.032 | -0.117,0.053 | -0.069 | 0.463 | 2.396 |
| Age | -5.442 | -8.624,-2.259 | -0.184 | ＜0.001 | 1.265 | -0.077 | -0.124,-0.03 | -0.222 | ＜0.001 | 1.265 |
| Smoking | -19.729 | -92.896,53.437 | -0.029 | 0.595 | 1.275 | -0.291 | -1.367,0.785 | -0.037 | 0.594 | 1.275 |
| HTN | 46.287 | -19.109,111.682 | 0.075 | 0.164 | 1.226 | 0.780 | -0.181,1.742 | 0.108 | 0.111 | 1.226 |
| DM | 25.820 | -26.742,78.381 | 0.063 | 0.334 | 1.786 | 0.366 | -0.407,1.139 | 0.076 | 0.351 | 1.786 |
| HbA1c | 6.540 | -34.488,47.567 | 0.022 | 0.754 | 2.049 | 0.099 | -0.504,0.702 | 0.028 | 0.747 | 2.049 |
| TG | -83.078 | -124.65,-41.506 | -0.234 | ＜0.001 | 1.492 | -1.155 | -1.767,-0.544 | -0.277 | 0.000 | 1.492 |
| HDL-C | -31.009 | -156.152,94.134 | -0.029 | 0.626 | 1.511 | -0.462 | -2.302,1.378 | -0.037 | 0.621 | 1.511 |
| Hb | 2.245 | -0.233,4.723 | 0.115 | 0.076 | 1.744 | 0.030 | -0.007,0.066 | 0.129 | 0.109 | 1.744 |
| LVEF | 6.253 | 1.117,11.389 | 0.122 | 0.017 | 1.089 | 0.083 | 0.007,0.158 | 0.137 | 0.032 | 1.089 |
| β-blocker use | -33.297 | -107.12,40.526 | -0.048 | 0.375 | 1.217 | -0.536 | -1.622,0.549 | -0.065 | 0.331 | 1.217 |
| Revascularization | -92.741 | -166.146,-19.336 | -0.149 | 0.014 | 1.509 | -1.367 | -2.446,-0.287 | -0.187 | 0.013 | 1.509 |
| Sex*BMI | -6.027 | -10.13,-1.923 | -0.234 | 0.004 | 2.760 | -0.093 | -0.153,-0.032 | -0.306 | 0.003 | 2.760 |
